# Analysis of the tendency for the electronic conductivity to change during alcoholic fermentation

**DOI:** 10.1038/s41598-019-41225-x

**Published:** 2019-04-02

**Authors:** Chongwei Li, Yue Wang, Shuang Sha, He Yin, Huilin Zhang, Yongsheng Wang, Bo Zhao, Fuqiang Song

**Affiliations:** 1 Engineering Research Center of Agricultural Microbiology Technology, Ministry of Education, Heilongjiang University, Harbin, 150500 China; 2 Heilongjiang Provincial Key Laboratory of Ecological Restoration and Resource Utilization for Cold Region, School of Life Sciences, Heilongjiang University, Harbin, 150080 China; 3 Department of Food Science and Engineering, School of Life Sciences, Heilongjiang University, Harbin, 150080 China

## Abstract

The observation that the electronic conductivity begins to decease and then increases during alcoholic fermentation was first discovered in our work. To explain the tendency experiments were conducted to investigate the effect of the reducing sugar concentration, ethanol concentration, cell density, pH and ionic concentration. The results showed that the ionic concentration, reducing sugar concentration, cell concentration, pH and especially the ethanol concentration caused a change of the electronic conductivity. From 0 h to 60 h, the ethanol concentration had a significant negative correlation with the conductivity, which decreased with increasing ethanol concentration during fermentation. From 60 h to 68 h, when the ethanol concentration remained unchanged, the total ionic concentration had a significant positive correlation with the electronic conductivity, which increased with increasing ionic concentration (pH value decreases, cell autolysis). Thus, when the electronic conductivity reached its lowest point, the alcoholic content was the greatest. We concluded that it is feasible to directly reflect the change of the ethanol concentration using the change of the electronic conductivity by constructing a mathematical model. The results of this model could be applied for the completely on-line monitoring of the alcoholic fermentation process and for determining the end point of fermentation.

## Introduction

Fermentation is the most important step of the alcoholic production process and directly affects the product quality and economic performance of modern enterprises^1,2^. To determine the end point of the fermentation process, taking samples from fermentation tanks to the laboratory and testing the sample in the laboratory has been the standard procedure. This off-line detection not only misses the most appropriate discharge time and prolongs the production cycle but also increases the consumption of manpower, materials and financial resources by measuring the ethanol concentration and reducing sugar once every 4 h^3,4^.

Researchers around the world have been committed to monitoring the dynamics of fermentation processes and determining the end of process using instruments and equipment^5–7^. For example, A unique liquid core light waveguide sensor was designed to monitor the ethanol concentration on-line^8^. Ultrasonic sensors for on-line monitoring of the ethanol concentration have been designed^9^. As well as, the electronic conductivity technology has been utilized as an index to reflect the fermentation process^10–12^.

**Figure 1:**
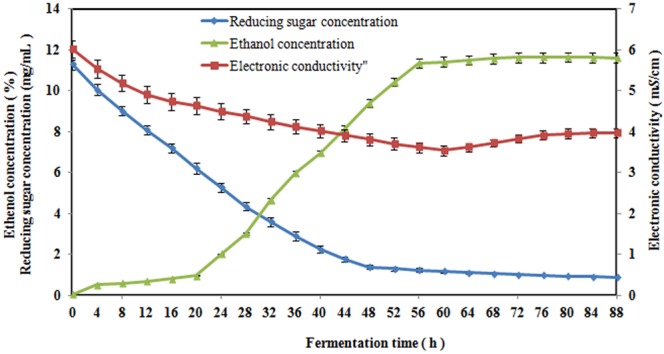
Changes of the electronic conductivity, ethanol concentration and reducing sugar concentration during the fermentation process. Error bars represent the standard error of mean of three replicates (n = 3).

Our previous studies have shown that there are changes of the electronic conductivity, ethanol concentration and reducing sugar concentration during a fermentation cycle (Fig. 1). From Fig. 1, we can see that during fermentation, the electronic conductivity decreased first and then increased, and the ethanol concentration gradually increased while the reducing sugar concentration decreased. However, it remains unknown why the electronic conductivity first shows a decreasing trend and then an increasing trend such that the lowest point of electronic conductivity is the end point of alcoholic fermentation. If this phenomenon can be understood, then we can obtain the ethanol concentration via the electronic conductivity using a mathematical model, which can serve as a theoretical basis for on-line monitoring of the alcoholic fermentation process^13,14^.

## Results

To explain the tendency for the electronic conductivity to change during alcoholic fermentation, this experiment considered the effect of the reducing sugar concentration, ethanol concentration, cell density, pH and ionic concentration on the change of the electronic conductivity during the alcoholic fermentation process.

### Effects of the ionic concentrations of K^+^, Na^+^, Ca^2+^ and Mg^2+^ on the electronic conductivity

Electronic conductivity is the ability to transfer current; it is the reciprocal of resistivity and is used to indicate the conductance of a solution^15^. A greater ionic concentration has a stronger electronic conductivity in solution, i.e., a greater electronic conductivity value. The concentrations of K^+^, Na^+^, Ca^2+^ and Mg^2+^ during different stages (12, 24, 36, 48, 56, 58, 60, 62 and 64 h) of the alcoholic fermentation process were measured using ICP-OES (Inductively Coupled Plasma Optical Emission Spectrometer).

The results showed that the concentration of the 4 ions in fermentation liquid had a significantly increasing trend from initial stage to 62 h (p < 0.05, one-way ANOVA). Specifically, at 62 h the final concentration of K^+^, Na^+^, Ca^2+^ and Mg^2+^ was increased by 76.84%, 94.09, 420% and 134.17% compared with the initial stage, respectively. The concentration change of Ca^2+^ was most obvious and the concentration of Mg^2+^ always is low level in whole fermentation process (Table 1). During this time period, the electronic conductivity decreased, showing a negative correlation with the ionic concentration, and then increased, showing a positive correlation with the ionic concentration.

**Table 1:** Concentration of four ions at different times. Different letters indicate significantly different values at *P* < 0.05.

Fermentation Time (h)	ionic concentration (mg/L)			
	K^+^	Na^+^	Ca^2+^	Mg^2+^
12	204.51 ± 23.43b	156.61 ± 16.32a	63.13 ± 6.67a	23.38 ± 2.16a
24	160.71 ± 17.86a	189.71 ± 18.65b	108.91 ± 10.12c	25.37 ± 2.32b
36	218.21 ± 25.63c	204.31 ± 23.32c	98.99 ± 9.98b	27.36 ± 2.67c
48	221.92 ± 27.34c	207.98 ± 24.56c	111.21 ± 11.67c	29.77 ± 3.01d
56	267.48 ± 28.01d	215.74 ± 25.34d	218.23 ± 22.01d	41.78 ± 3.98e
58	319.67 ± 31.32e	224.91 ± 27.76e	257.45 ± 24.78e	45.67 ± 4.34f
60	337.84 ± 33.23f	254.84 ± 27.86f	272.26 ± 27.12f	48.58 ± 4.65g
62	361.66 ± 35.12g	303.96 ± 30.56g	329.08 ± 31.78g	54.75 ± 5.23h

### Effect of pH on the electronic conductivity

**Figure 2:**
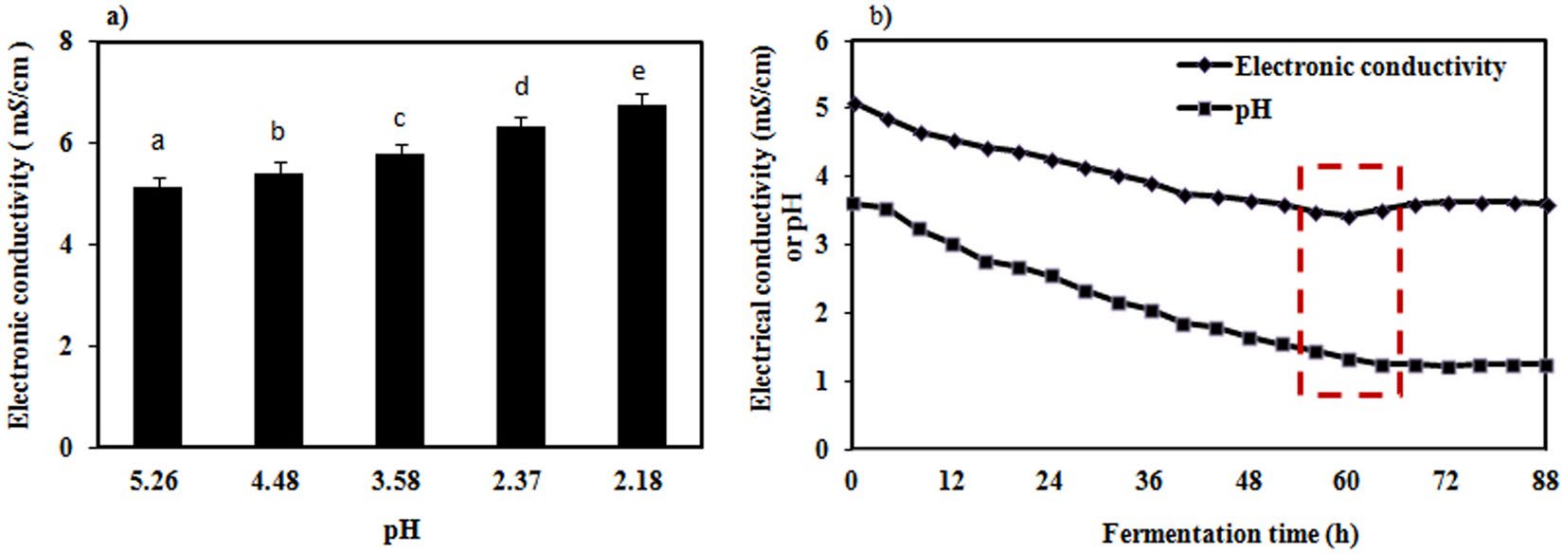
Relationship between the pH and the electronic conductivity (**a**) and the relationship between the pH and the electronic conductivity during fermentation (**b**). Different letters indicate significantly different values at *P* < 0.05. Error bars represent the standard error of mean of three replicates (n = 3).

Alcoholic fermentation is a process in which glucose is gradually decomposed to produce alcohol and other acidic substances, which causes an increase in the ionic concentration of H^+^ ^16^. In this experiment, the electronic conductivity and pH value during the fermentation process were measured every 4 h, and the electronic conductivity in inorganic salt solutions at different pH values were also measured (Fig. 2). In Fig. 2a, a negative relationship between the pH value and the electronic conductivity can be observed; specifically, the pH decreased from 4.48 to 1.84, and the electronic conductivity increased gradually from 5.43 m*S*/cm to 8.26 m*S*/cm. These results agreed with the basic definition of conductivity (Fig. 2a).

During the alcoholic fermentation process, from 0 h to 60 h, the electronic conductivity and pH significantly decreased (p < 0.05, one-way ANOVA), i.e., the pH value decreased from 3.61 to 1.24 (Fig. 2b) while the electronic conductivity decreased from 5.11 m*S*/cm to 3.42 m*S*/cm (Fig. 2b); these results agree with the result in Fig. 2a. From these results, we inferred that the decrease of the electronic conductivity was not caused by the pH. From 60 h to 64 h, the pH continued to decrease and decreased by 32.57%, and the electronic conductivity stopped decreasing and then began increasing, with an increase of 28.69%. From 64 h to 68 h, the pH and electronic conductivity stabilized (Fig. 2b), from which we infer that the pH change influenced the change of the electronic conductivity when the ethanol concentration was constant.

### Effect of the reducing sugar concentration on the electronic conductivity

**Figure 3:**
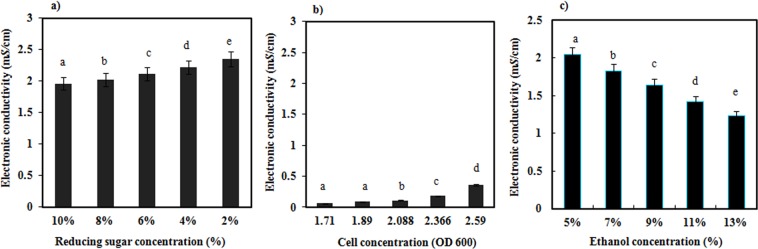
Relationship between different reducing sugar concentrations and the electronic conductivity (**a**); relationship between different cell concentrations and the electronic conductivity (**b**); relationship between different ethanol concentration and the electronic conductivity (**c**). Different letters indicate significantly different values at P < 0.05. Error bars represent the standard error of mean of three replicates (n = 3).

We designed an experiment in which the electronic conductivity and reducing sugar concentration were measured in solutions with different amounts of added glucose^17^. The results showed that the reducing sugar concentration decreased from 10% (w/v) to 2% (w/v), and the conductivity increased from 1.96 m*S*/cm to 2.35 m*S*/cm. There was a negative correlation between these measures (Fig. 3a). In the actual fermentation process, as the reducing sugar concentration decreased, the electronic conductivity decreased as well. Therefore, we inferred that the reducing sugar concentration was not the main factor causing the electronic conductivity to decrease.

### Effect of the cell density on the electronic conductivity

Cells have a negative charge; therefore, a change in the cell number may lead to a change of the electronic conductivity in solution. Single-factor experiments of the cell concentration and the electronic conductivity showed that as the cell density increased, the electronic conductivity increased from 0.06 mS/cm to 0.36 m*S*/cm (Fig. 3b). However, during actual alcoholic fermentation, as the cell density increased in the fermentation medium, the electronic conductivity decreased. Therefore, we inferred that the increase in cell density was not the main factor causing the change in the electronic conductivity.

### Effects of the ethanol concentration on the electronic conductivity

To investigate the influence of the ethanol concentration on the electronic conductivity, the relationship between the ethanol concentration and the electronic conductivity in solutions with different ethanol concentrations was determined. A negative relationship between the ethanol concentration and electronic conductivity was observed; as the ethanol concentration increased from 5% (v/v) to 15% (v/v), the electronic conductivity decreased from 2.04 m*S*/cm to 1.05 m*S*/cm (Fig. 3c), which agrees with the change in the ethanol concentration and electronic conductivity. We can see that the change of the ethanol concentration in the alcoholic fermentation from 0 h to 60 h was the only experiment to correlate with the electronic conductivity in the single-factor experiments. Although the electronic conductivity did not decrease as much as in the actual process of alcoholic fermentation (the actual reduction was 1.5 m*S*/cm and also depended on other factors in solution), one could say that there was a direct relationship between the increase of the ethanol concentration and the decrease of the electronic conductivity. Subsequently, as the ethanol concentration remained basically unchanged, the electronic conductivity ceased its downward trend during the alcoholic fermentation process.

### Effect of cell death on the electronic conductivity

**Figure 4:**
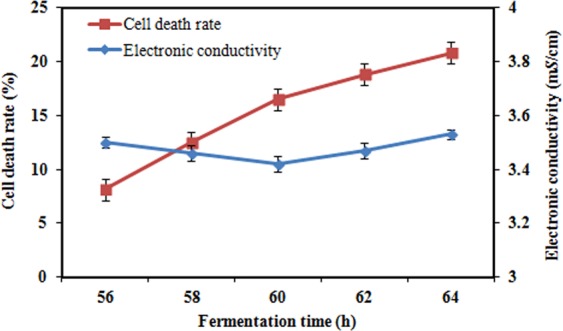
The cell death rate during the fermentation process at 56, 58, 60, 62 and 64 h. Error bars represent the standard error of mean of three replicates (n = 3).

**Figure 5:**
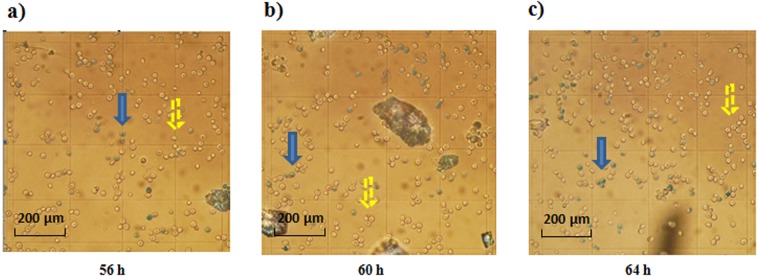
Cell morphology during the fermentation process at 56 h (**a**), 60 h (**b**) and 64 h (**c**). (blue arrows indicates living cells; yellow arrows indicates dead cells).

In the late stage of fermentation, cells began to be disrupted by the increase of the ethanol content, and large amounts of intracellular electrolytes entered the fermentation liquid. To investigate the effect of cell death on the electronic conductivity before and after 60 h, we measured the cell mortality at 56, 58, 60, 62 and 64 h (Fig. 4). The cell morphology was imaged using microscopy (Fig. 5). Yeast cell mortality increased from 8.98% to 20.87% from 56 h to 64 h (Fig. 4), and the mortality rate increased linearly (R^2^ = 0.9963). This result indicates that the yeast cell death in the late fermentation was gradual. There was an increasing number of assayed cells that were dyed blue in the fermentation from 56 h to 64 h, indicating that the number of dead yeast cells was growing linearly, but changes of the cell morphology at 60 h were not obvious (Fig. 5). We used the Three Chloroacetic Acid disruption Method (TCA Method. We compared the Ultrasonic disruption Method, Glass bead disruption Method, TCA disruption Method, Finally, the TCA disruption Method was chosen.) to break cells, which caused the electrical conductivity changed from 3.94 m*S*/cm (before breaking) to 5.98 m*S*/cm (after breaking) and the obvious deformation of cells observed using the microscope, this result again shows the cell death is gradual and linearity (Fig. 4).

Ergosterol is an important component of the fungal cell membrane structure. Ergosterol deficiency causes fungal cell membrane dysfunction and even cell rupture. These events will cause the formation of lipid peroxidation products such as malondialdehyde (MDA); transform the fluidity and permeability of the cell membrane; and change the structure and function of the cells.

**Figure 6:**
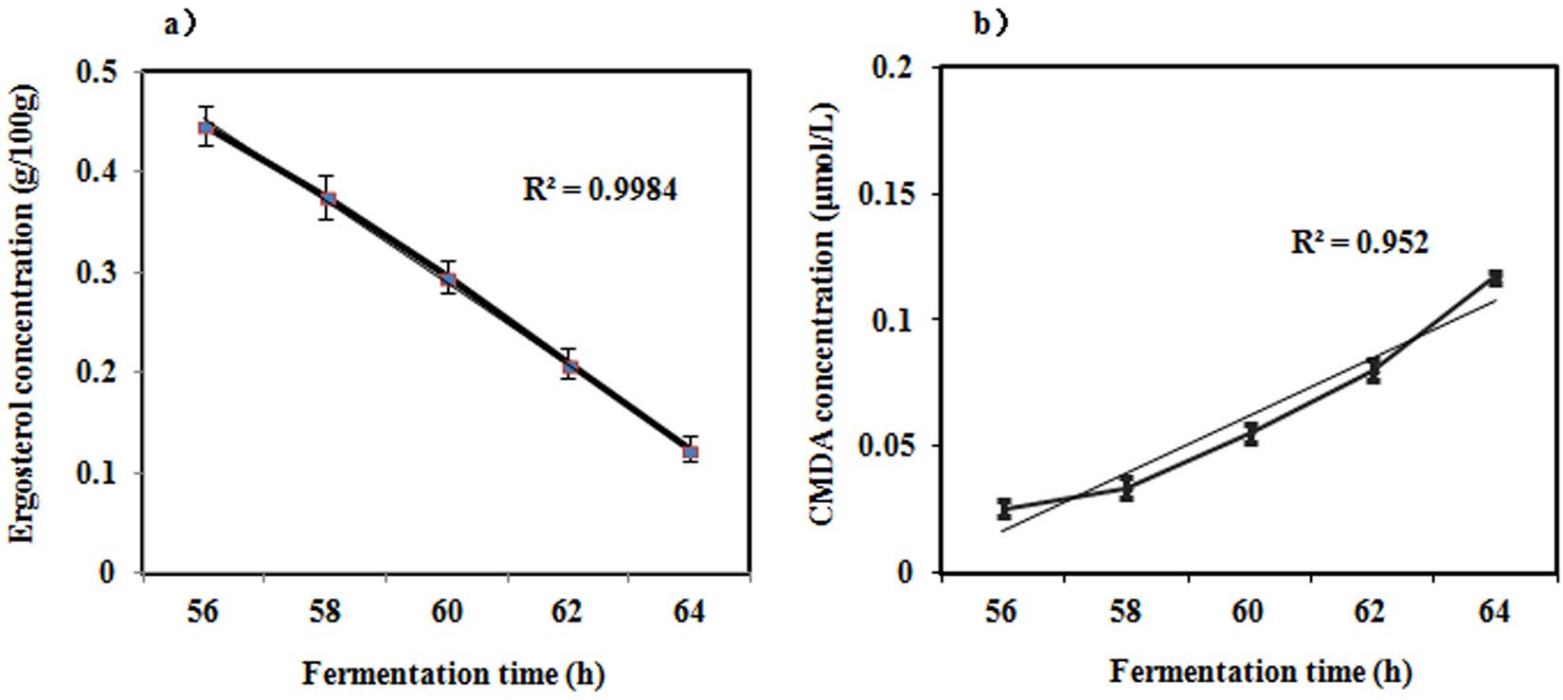
Ergosterol concentration (**a**) and MDA concentration (**b**) at different fermentation times. Error bars represent the standard error of mean of three replicates (n = 3).

The decrease in the ergosterol concentration from 0.446% (g/100 g) to 0.124% (g/100 g) and the increase in the MDA concentration from 0.026 μmol/L to 0.119 μmol/L at 56 h to 64 h, and the R^2^ values from both processes were greater than 0.95, indicating that the cell death rate changed linearly before and after 60 h, which further verified that the structure of the cell membrane with cell death was changed, and the permeability of the cell membrane increased at this stage (Fig. 6). Ultimately, from 60 h to 64 h, the electronic conductivity increased due to the ionic outflow under the ethanol concentration remained unchanged in the solution (Fig. 6).

### The mathematical model prediction

**Figure 7:**
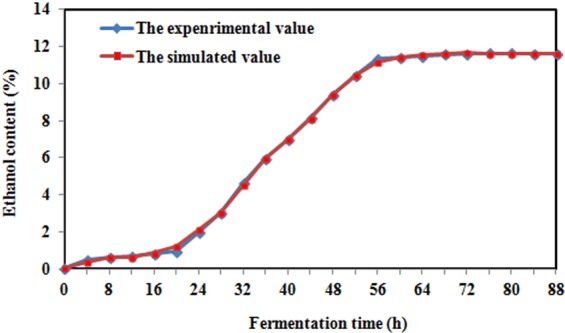
Profiles of both the simulated and the experimental ethanol content.

Back propagation is the most widely applied and effective machine learning method^18^. By taking the pH value, glucose concentration and cell concentration as input parameters and taking the ethanol concentration as the output parameter, a simulated ethanol concentration curve was obtained using a neural network. Figure 7 shows the simulated ethanol concentration. The maximum error between the simulated value and the experimental value was 14.8%.

## Discussion

In recent years, questions regarding on-line monitoring and determining the end point of alcoholic fermentation processes have become hot topics. An innovative and novel study of this problem utilizing the electronic conductivity is reported in this paper. From 0 h to 60 h, although the changes of the ionic concentration, reducing sugar concentration, pH and cell concentration can all lead to an increase of the electronic conductivity, under the condition of an increasing ethanol concentration, the effects of these four factors are counteracted, and the electronic conductivity decreases continuously, which result shows that increasing of ethanol concentration was the decisive factor for the decreasing electronic conductivity, which is shown in Fig. 1. This result should be related to the non-electrolytic nature of alcohol^19^. In a mixture of ethanol and water, with increasing ethanol concentration, the number of hydrogen bonding interactions between alcohol and water increases, which reduces the amorphous region in the solution, reduces the speed of the ions in the solution, and ultimately leads to a decrease in the electronic conductivity^20,21^.

At the lowest electronic conductivity value, the rate of change in the reducing sugar concentration and ethanol concentration was close to zero, which indicates that the alcoholic fermentation ended. Therefore, we can determine the end of alcoholic fermentation using the change in the electronic conductivity. To the best of our knowledge, this conclusion had not previously been reported at home or abroad.

After 60 h, the ethanol concentration remained unchanged, and the reducing sugar was consumed, and ionic concentration increased, which certainly included K^+^, Na^+^, Ca^2+^, Mg^2+^ and H^+^. The rate of change of the H^+^ concentration was negatively correlated with the rate of change of the electronic conductivity at 60 h to 64 h (Fig. 2b), the pH and the electronic conductivity tended to be stable from 68 h (Fig. 2b). Therefore, the ion outflow from the dead cells and a decrease of the pH were the main reasons for the increase of the electronic conductivity from 60 h.

In summary, from 0 h to 60 h, the electronic conductivity decrease was due to the continual increase of the ethanol concentration; from 60 h to 88 h, the electronic conductivity stopped deceasing and began to increase because the ethanol concentration remained unchanged in the fermented liquors, and the ionic concentration gradually increased. Therefore, the lowest point of the transformed process of the electronic conductivity represented the end of alcoholic fermentation. This finding can be used both to determine the end of alcoholic fermentation in a timely manner and to reflect the change of the ethanol concentration, and can be applied to obtain whether the fermentation process was normal or not. The application of this finding could be of great significance for the online monitoring of the fermentation process.

## Methods

### Alcoholic fermentation experiment design

The ratio of corn flour and water was 1:3.5 (g/g). The liquefying conditions were the following: 60 °C for 0.5 h and 100 °C for 90 min, with a pH of 6.0. The saccharification was at 60 °C for 30 min with a pH at 4.0. The must was inoculated with yeasts at 3%. The fermentation time was 88 h (30–32 °C for 0–12 h, 32–34 °C for 12–48 h and 30–32 °C for 48–88 h). The fermentation was carried out a 3 L stainless steel fermentation tank (Bioengineering AG, Switzerland), which was stirred before each sample was obtained. The electronic conductivity, the reducing sugar concentration and the ethanol concentration were tested every 4 h during the fermentation process. The reducing sugar was determined using 3,5-dinitrosalicylic acid method^22^. The electronic conductivity was measured using an EC-214 electronic conductivity meter (Shanghai Precision Instruments Co., Ltd., CHN). The ethanol concentration was measured by distillation^23^.

### Determination of the ionic concentration

Electronic conductivity is the reciprocal of resistivity and is used to indicate the conductance of a solution. To explain the effect of ionic concentration in the fermentation broth on the electronic conductivity, the ionic concentration during the alcoholic fermentation process was determined by measuring the concentrations of K^+^, Na^+^, Ca^2+^ and Mg^2+^ in the fermentation broth using Inductively Coupled Plasma Optical Emission Spectroscopy (ICP-OES) with an ICP-Optima 7000DV (Perkin Elmer, USA) system^24^. The instrument parameters are listed below (Table 2).

**Table 2:** ICP Experimental Parameters.

Element	Wavelength (nm)	Plasma gas flow rate (L/min)	Auxiliary gas flow (L/min)	Nebulizer gas flow (L/min)	RF power (w)	Peristaltic pump flow (mL/min)
K	766.49	15	0.2	0.8	1300	1.5
Na	589.592	15	0.2	0.8	1300	1.5
Ca	317.933	15	0.2	0.8	1300	1.5
Mg	285.213	15	0.2	0.8	1300	1.5

### Determination of the pH and electronic conductivity

To explain the effect of ionic concentration of H^+^ in the fermentation broth on the electrical conductivity during the whole fermentation process, the relationship between the electronic conductivity and pH were determined every 4 h during the fermentation. The pH was measured using an FE20-FiveEasy pH meter (Beijing United Tech Co. Ltd., CHN), and the electronic conductivity was measured using an EC214 electronic conductivity meter.

### Determination of the reducing sugar concentration and electronic conductivity

To explain the effect of the reducing sugar concentration in the fermentation broth on electrical conductivity during the whole fermentation process, the relationship between the reducing sugar concentration and the electronic conductivity were determined for solutions containing 0.1% NaCI with a final reducing sugar content of 10%, 8%, 6%, 4% and 2%.

### Determination of the ethanol concentration and electronic conductivity

To explain the effect of the ethanol concentration in the fermentation broth on electrical conductivity during the whole fermentation process, the relationship between the ethanol concentration and the electronic conductivity were determined for solutions containing 0.1% NaCI with an ethanol content of 5%, 7%, 9%, 11%, 13% and 15%.

### Determination of the cell density and electronic conductivity

To explain the effect of the cell concentration in the fermentation broth on electronic conductivity during the whole fermentation process, the relationship between the cell concentration and the electronic conductivity were determined for solutions containing 0.9% NaCI with different cell concentrations by diluting yeasts in the logarithmic growth phase (8.2 × 10^6^ CFu/mL) 0, 2, 4, 6, and 8 times with a sterile saline solution (0.9% NaCI), and the different OD values were tested. The OD values were measured using the turbidity method.

Determination of the cell mortality, ergosterol and malondialdehyde concentration. The mortality, ergosterol concentration^25^ and malondialdehyde (MDA)^26^ concentration of the yeast cells in the fermentation broth were determined at 56, 58, 60, 62 and 64 h. The morphological changes of the cells were observed by microscope.

### Cell disruption method

We attempted to break down yeast cells using the TCA Method and to determine the electronic conductivity with the cells obtained by diluting the yeast in the logarithmic growth phase (8.2 × 10^6^ CFu/mL). In brief, a TCA disruption Method was the following: 4.5 mL of Three chloroacetic acid was added to 50 mL of the cell suspension solution; then, the mixture was placed in an ice bath for 30 min^27^.

### The establishment and prediction of mathematical models

Back propagation is the most widely applied and effective machine learning method. BP network is composed of an input layer, a hidden layer and an output layer. The number of units in the input and output layer is determined by the number of specific problems. The number of units in the hidden layer is determined by the complexity and the error reduction of the specific problem^28,29^.

**Figure 8:**
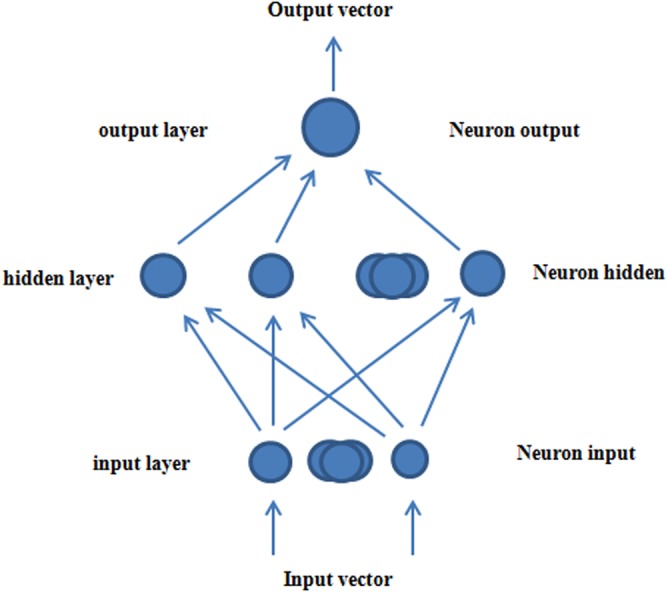
Figure of BP neural network model.

The BP algorithm with an impulse term is used in the learning algorithm of the neural network model (Fig. 8).

(1) Input learning samples

       Input vector Xp, (p = 1, 2, …, P) and target output Tp, (p = 1, 2, …, P)

(2) The actual output of the network and the state of the implicit units

       O_PJ_ = F_J_ (∑ − ƜπO_I_ − θ_J_)

There are the following:

                   O–Neuron output

                   Ɯ–weight value

                   Ɵ–the thresholds of neurons

The excitation function F is a sigmoid function,

            (X) = 1/[1 + exp (−X)]

(3) raining error output layer

input layer Ϭ_PJ_ = O_PJ_ (1 − O_PJ_) (t_PJ_ − O_PJ_)

output layer Ϭ_PJ_ = O_PJ_ (1 − O_PJ_) ∑Ϭ_PK_Ɯ_JK_

(4) Modify weights and thresholds

           Ɯπ (t + 1) = Ɯπ (t) + ƞϬ_J_O_PJ_ + α [Ɯπ (t) − Ɯπ (t − 1)]

There are the following:

                     ƞ–Learning pace, this model uses 0.5

                     α–dynamic item, this model uses 0.5

After P reaches 1−P, judge whether the index fulfils the accuracy requirement E, E < ɛ and whether the following applies: E = ∑EP, EP = ∑(t_PJ_ − O_PJ_) − 2/2, where ɛ is accuracy and ɛ = 0.0001. When the results have satisfied the requirements, the operation is stopped; otherwise, the operation is repeated.

By taking the pH, glucose concentration and cell concentration as input parameters and by taking the ethanol content as the output parameter, 30 groups of data were used for network training in the process of network modelling. There were 8 hidden layers, and the other 7 groups of data were used as validation values so that the weight value of the fuzzy comprehensive evaluation index gradually approached the actual situation. The simulated ethanol content curve of the network was obtained, and the simulated value was compared with the experimental value. Matlab (MathWorks Co. Ltd., USA) software was used for the mathematical models establishment.

### Data Processing

SPSS 11.5 software (Chicago, IL USA) was used for statistical analysis, and a t-test was used for significant difference analysis. P < 0.05 was considered significant.
